# 3D models for melanoma γδ T cell‐based immunotherapy

**DOI:** 10.1002/ctm2.926

**Published:** 2022-06-13

**Authors:** Veronica Huber, Viviana Vallacchi, Elena Daveri, Elisabetta Vergani

**Affiliations:** ^1^ Department of Research Unit of Immunotherapy of Human Tumors Fondazione IRCCS Istituto Nazionale dei Tumori Milan Italy

**Keywords:** γδ T cells, melanoma 3D models, immunotherapy, epigenetic modifiers

1

Immunotherapy, including immune checkpoint inhibitors (ICI), induces durable clinical responses in multiple human malignancies, as for melanoma, but the number of responding patients remains globally modest. The limited therapeutic efficacy of ICI depends on multiple factors, among which the immune suppressive features of the tumor microenvironment (TME) play a key role, and experimental models allowing access to the immune‐hostile tumor milieu components may unravel how to overcome resistance and obtain full‐fledged anti‐tumor immunity. The establishment of 3D platforms able to predict clinical responses would permit investigating the functional roles of specific cell types and soluble factors during ICI or combination therapies to assess existing correlations between clinical and immunological response, predictive immune response baseline parameters and subtypes of unresponsive tumors.^1^

In such a scenario, the new study by Ou et al.^2^ sheds light on the usefulness of 3D models as preclinical platforms to study the therapeutic efficacy of adoptive immunotherapy based on the human γδ T cells, non‐MHC‐restricted T cells emerging as a valid alternative to conventional αβ T cells in melanoma.^3^

γδ T cell‐based immunotherapy has been studied in solid tumors and hematological malignancies, but despite representing 15–25% of infiltrating cells as for melanoma, research investigating the immunotherapeutic potential of γδ T cells is still at preclinical level for this type of cancer.^3^ Higher frequencies of Vδ2 T cells in peripheral blood of melanoma patients are associated with increased overall survival,^4^ but their functional role in TME and their anti‐tumor potential are still unexplored due to the lack of adequate preclinical models. Several studies reported the capacity of T and NK cells of infiltrating and killing tumor spheroids,^5^, ^6^ but the information for γδ T cells is scant. In their study Ou et al.^2^ explore activation, tumor infiltration and killing activity of Vγ9Vδ2 T cells (also termed Vδ2 T cells) exogenously administered to different 3D platforms with increasing cellular complexity, i.e. unicellular melanoma spheroids, bicellular tumor‐fibroblast spheroids, multicellular patient‐derived spheroids and melanoma patient‐derived organoids (MPDOs). By applying the diverse models, they show that γδ T cells are more efficient than αβ T cells in migrating and infiltrating melanoma spheroids, as well as the other 3D models; however, the exposure to inflammatory and immunosuppressive signals delivered by the TME, readily induces CTLA‐4, PD‐1 and PD‐L1 upregulation, which leads to γδ T cell exhaustion, block of antitumor cytolytic activity and polarization into tumor‐promoting effectors, thus contributing to melanoma progression.^2^, ^7^

These data clearly indicate that, when facing TME, activated effectors undergo a hypo‐responsive state that limits their functional properties and significantly impacts the initial strength of immune cells. Indeed, when CTLA‐4 and PD‐1 ICI in combination are also added to the co‐cultures, strong IFNγ production, potentiated γδ T cell infiltrate and immune‐mediated tumor killing are detected. However, as also underlined by Ou et al.,^2^ not all 3D models perform equally and may faithfully reproduce what occurs at patient's tumor site. Indeed, MPDOs are the only ones to represent native TME, with the presence of diverse antitumor as well as protumor factors, and the multiple and complex immune cell components. Spheroid/MPDO models also suited the testing of drugs that target epigenetic mediators sustaining immunosuppressive TME and their impact on reversing exhaustion, to ultimately restore anti‐tumor activity of γδ T cells.^2^ In particular, Ou et al. demonstrate that the epigenetic modifiers Entinostat and Vorinostat enhanced γδ T cell cytotoxicity and chemotaxis against MPDOs, through the downregulation of PD‐1, upregulation of CXCR3/CD107a expression, IFNγ production in combination with the MICA/B upregulation and PD‐L1 downregulation in melanoma cells.^2^

In sum, the results of the work by Ou et al.^2^ provide an important rationale for γδ T cell‐based immunotherapy to overcome immunotherapy resistance in melanoma patients. The authors encourage the establishment in the clinics of personalized ex vivo platforms to test immunotherapy efficacy, define prediction markers and develop combination strategies. Increased investment in this promising type of research could contribute to the design of optimal personalized treatments aimed at preventing or overcoming resistance onset in a patient‐tailored manner, to ultimately improve clinical outcome.

## CONFLICT OF INTEREST

The authors declare no conflict of interest.
